# Supplementary material for: Evaluating Habitat Suitability for the Endangered Sinojackia xylocarpa (Styracaceae) in China Under Climate Change Based on Ensemble Modeling and Gap Analysis
Source: Biology (Basel). 2025 Mar 17;14(3):304. doi: 10.3390/biology14030304 (PMC11940633; doi:10.3390/biology14030304)
Supplement: Supplementary file 1 [file biology-14-00304-s001.zip › biology-3491002-supplementary.pdf]

Table S1. Latitude and longitude coordinates of 21 occurrence records of the endangered *Sinojackia xylocarpa* in China.

No.	Species	Longitude(°)	Latitude(°)
1	*Sinojackia xylocarpa*	112.3536	28.8487
2	*Sinojackia xylocarpa*	112.7254	29.1870
3	*Sinojackia xylocarpa*	113.9125	30.5352
4	*Sinojackia xylocarpa*	121.2726	30.1753
5	*Sinojackia xylocarpa*	118.8020	32.1292
6	*Sinojackia xylocarpa*	114.6256	31.2958
7	*Sinojackia xylocarpa*	114.6468	31.2533
8	*Sinojackia xylocarpa*	118.8098	32.1402
9	*Sinojackia xylocarpa*	119.0785	32.1404
10	*Sinojackia xylocarpa*	118.6078	32.1066
11	*Sinojackia xylocarpa*	110.9125	30.0117
12	*Sinojackia xylocarpa*	109.8478	27.6722
13	*Sinojackia xylocarpa*	110.0760	28.6694
14	*Sinojackia xylocarpa*	109.4886	28.5779
15	*Sinojackia xylocarpa*	114.5833	29.3752
16	*Sinojackia xylocarpa*	116.6222	29.7420
17	*Sinojackia xylocarpa*	115.8015	28.6577
18	*Sinojackia xylocarpa*	116.0011	29.5615
19	*Sinojackia xylocarpa*	117.5877	31.1281
20	*Sinojackia xylocarpa*	118.4850	29.9185
21	*Sinojackia xylocarpa*	116.0199	29.9986
